# Feasibility and acceptability of a mindfulness-based vitality promotion program in a lifelong learning setting: a single-arm mixed-methods study

**DOI:** 10.3389/fpubh.2026.1990679

**Published:** 2026-10-28

**Authors:** Seok-In Yoon, Hui-Yeong Park, Jae-Hong Yoo, Sun-Yong Chung, Jong Woo Kim

**Affiliations:** 1 Department of Neuropsychiatry, College of Korean Medicine, Kyung Hee University, Seoul, Republic of Korea; 2 Department of Neuropsychiatry, Kyung Hee University Korean Medicine Hospital at Gangdong, Seoul, Republic of Korea

**Keywords:** feasibility, lifelong learning, mindfulness, mixed-methods study, public health, vitality

## Abstract

**Background:**

As global aging accelerates, age-related psychophysiological conditions have emerged as major public health concerns. Promoting and sustaining vitality, an inherent human capacity, is essential for healthy aging and long-term well-being. The Mindfulness-based Vitality Promotion (MVP) program is a mindfulness-based behavioral intervention designed to enhance physical and psychological vitality and support sustainable well-being. This study evaluated the feasibility and acceptability of the MVP program and explored preliminary pre–post changes in health-related outcomes among community-dwelling individuals in a lifelong learning setting.

**Methods:**

This study employed a single-arm mixed-methods design. A total of 35 participants were recruited for this study. The MVP program was delivered over 8 weeks, with each session lasting 90 min. Quantitative data were collected through self-report questionnaires, and qualitative data were obtained via semi-structured interviews. Feasibility and acceptability were assessed based on program retention, session attendance, and participant satisfaction.

**Results:**

Quantitative analysis revealed significant pre–post increases in integrative vitality and well-being, along with significant reductions in depression, fatigue, and anxiety. No statistically significant changes were observed in pain, sleep disturbance, digestive symptoms, anger, or mindfulness. Of the 35 enrolled participants, 31 completed the program (88.6%), with a mean attendance of 6.37 of eight sessions. Among the 30 satisfaction survey respondents, 28 (93.3%) indicated willingness to participate again and 30 (100%) indicated willingness to recommend the program to others. The qualitative analysis identified four major themes: (i) diverse life challenges and self-care needs, (ii) learning through experience, connection, and application, (iii) regaining balance and agency in life, and (iv) sustaining practice requires continued support.

**Conclusion:**

Mixed-methods findings provide preliminary information regarding the feasibility and acceptability of the MVP program and indicate favorable pre–post changes in several self-reported health-related outcomes. The MVP program may represent a practical lifelong learning intervention with potential relevance for public health promotion. Further controlled studies are warranted to establish its efficacy.

**Trial registration:**

The trial was first submitted to the Clinical Research Information Service, Republic of Korea (KCT0009961, https://cris.nih.go.kr/cris/search/detailSearch.do?seq=28524&search_page=L), on October 8, 2024, before participant enrollment began, and registration was finalized on November 28, 2024.

## 1 Introduction

Population aging has emerged as a major global public health concern, particularly in developed and developing countries (1). In 2022, the global proportion of adults aged ≥ 65 years was 9.7%, which is projected to increase to 11.7% by 2030 and 16.4% by 2050 (2). In Korea, the proportion of older adults has increased rapidly, reflecting the country’s rapid population aging. The aging rate in Korea is increasing more rapidly than that in European countries, where population aging began earlier. This trend is expected to accelerate further owing to declining birth rates.

With the growing aging population, the physical and mental health challenges associated with aging, such as frailty, chronic diseases, and depression, have become critical public health issues. These conditions are commonly associated with depletion of the physical and emotional resources required to cope with stressors. The socioeconomic burden of age-related diseases is increasing, highlighting the need for a shift in the health paradigm toward prevention and management (1). Approximately 58% of the population aged ≥ 50 years have been identified as frail or pre-frail (3). Notably, frailty, chronic diseases, and depression are not independent risk factors; the presence of one can increase the risk of developing others. Previous studies have shown that frailty reduces stress resistance, thereby increasing vulnerability to chronic diseases and depression (4, 5). Meanwhile, depression is a known risk factor for chronic diseases, including heart disease and stroke (6). Among individuals with chronic conditions, the prevalence of depression tends to increase in parallel with these conditions (7). These findings suggest that a multidimensional approach, encompassing physical, psychological, and social domains, is essential for effective health management in later life.

Aging is not merely a matter of chronological age but a lifelong process involving progressive declines in physical and psychological functioning. Therefore, maintaining functional capacity throughout the life course is important for both older and younger populations. Over the past decade, the prevalence of chronic diseases has significantly increased in younger populations (8). In parallel, depression remains a major global mental health concern, with approximately 330 million prevalent cases estimated worldwide in 2021 (9). The early onset of chronic diseases is a risk factor for depression and frailty later in life (7, 10). Therefore, early prevention and management of functional decline are essential for maintaining health and well-being throughout the aging process.

Vitality represents an important capacity that should be promoted and maintained throughout the life course to support healthy aging. Healthy aging refers to the process of developing and preserving the functional ability that enables well-being in older age (11). According to the World Health Organization (WHO), healthy aging can be achieved by developing and maintaining five intrinsic capacities: mobility, cognition, sensory function, psychological well-being, and vitality (12). One of the WHO’s definitions of vitality is the “capacity to retain capacity” (12). This definition suggests that vitality may serve as a foundational capacity that supports the maintenance of other intrinsic capacities by promoting homeostasis and resilience.

Historically, vitality has been understood as the inherent energy of living organisms. However, in modern times, it can be assessed using objective indicators, such as vital signs, grip strength, and walking speed (13–17). In psychological and health research, vitality has also been conceptualized and assessed as a subjective and multidimensional experience involving physical and psychological aspects (18–20). Subjective vitality, which refers to the subjective experience of feeling alive and energetic (18), has been associated with greater resilience to psychological stressors (21, 22). A related but distinct construct, emotional vitality, reflecting positive psychological functioning that includes positive energy and well-being, has been prospectively associated with a reduced risk of coronary heart disease (23) and adverse health outcomes, including mortality (24). In the present study, vitality refers specifically to integrative vitality, a self-reported construct encompassing physical and psychological vitality as operationalized by the Integrative Vitality Scale (IVS) (20).

Mindfulness refers to an intentional state of awareness focused on the present moment with a non-judgmental attitude (25, 26). Previous studies have demonstrated that mindfulness enhances the ability to manage and accept chronic pain (27), improves sleep disorders (28), prevents relapse of depression (29), and increases subjective vitality (30). Collectively, these findings suggest that mindfulness may be relevant to multiple dimensions of health and well-being, including both physical and psychological aspects.

Lifelong learning provides a potentially valuable setting for promoting health and well-being within communities (31). It has been defined as “all learning activities undertaken throughout life, to improve knowledge, skills and competences within a personal, civic, social and/or employment-related perspective” (32). Previous studies have shown that participation in lifelong learning improves perceived health and well-being (33, 34). In-depth interviews have shown that lifelong learning improves participants’ ability to cope with stress, enhances resilience, and improves well-being (35). These findings suggest that participation in lifelong learning may have broader relevance for health and well-being at the community level (33–35).

Based on this framework, we developed the Mindfulness-based Vitality Promotion (MVP) program to enhance physical and psychological vitality and support sustainable well-being and healthy aging. Established mindfulness-based programs, such as Mindfulness-Based Stress Reduction (MBSR) and Mindfulness-Based Cognitive Therapy (MBCT), were originally developed to address clinical concerns such as chronic pain and depression relapse, respectively. By comparison, MVP adopts a health promotion-oriented approach with potential applications in community-based public health settings.

This study primarily aimed to examine the feasibility and acceptability of implementing the MVP program in a community-based lifelong learning setting, focusing on program retention, session attendance, and participant satisfaction. In addition, exploratory pre–post analyses were conducted to examine changes in health-related outcomes. Among these outcomes, overall integrative vitality was the primary outcome, while physical and psychological vitality were examined as domain-specific exploratory outcomes; the remaining health-related outcomes were also considered exploratory. Semi-structured interviews were conducted to gain an in-depth understanding of participants’ experiences, perceived benefits, and challenges associated with the MVP program.

## 2 Methods

### 2.1 Study design

This study employed a single-arm, sequential mixed-methods design with quantitative priority. The study was conducted at a public lifelong learning institution operated by the Seoul Metropolitan Institute for Lifelong Education in Seoul, Republic of Korea. Participants were recruited through an official program announcement posted on the institution’s website. Individuals were provided with detailed information about the study, including its purpose and procedures, and those who voluntarily agreed to participate were enrolled in the study. Written informed consent was obtained from all participants before eligibility screening.

Quantitative data included feasibility and acceptability indicators and self-report measures collected before and after the 8-week intervention, while qualitative data were obtained through semi-structured interviews to explore participants’ experiences and perceptions of the program. The quantitative component was used to assess feasibility and acceptability and to examine pre–post changes in health-related outcomes, whereas the qualitative component provided complementary information on participants’ experiences, perceived changes, and challenges associated with the program. The two components were integrated during the interpretation and reporting of the findings.

This study was reported in accordance with the applicable items of the Transparent Reporting of Evaluations with Nonrandomized Designs (TREND) statement and the Consolidated Criteria for Reporting Qualitative Research (COREQ) guidelines (Supplementary material 1).

### 2.2 Participants

A minimum sample size of 30 participants was initially planned for the quantitative analysis and was specified in the trial registration. Given the preliminary feasibility nature of the study, this sample size was pragmatically determined rather than based on a formal power calculation. To account for an anticipated dropout rate of approximately 20%, the target enrollment was subsequently increased to 38 participants, with IRB approval obtained on December 29, 2024. After completion of the final group (Group C), 31 participants had completed the program, exceeding the initially planned minimum sample size of 30. Therefore, no additional participants were recruited.

Participants were eligible if they (i) were adults aged ≥19 years; (ii) experienced any physical or psychological discomfort, such as depression, fatigue, frailty, or stress; and (iii) were able to participate in the 8-week program. Physical or psychological discomfort was assessed based on participants’ self-report during eligibility screening; no diagnostic or symptom-severity threshold was applied. No specific exclusion criteria were applied, and no additional clinical assessment or referral procedures were implemented based on clinical symptom severity. A total of 35 eligible participants were enrolled in the study.

The criteria for dropping out were as follows: (i) missing four or more sessions of the MVP program, (ii) voluntary withdrawal due to physical or psychological discomfort during the study, and (iii) voluntary withdrawal for other reasons. No separate systematic procedure for assessing adverse events was implemented. Participants were permitted to discontinue participation if they experienced physical or psychological discomfort during the study.

### 2.3 Procedure

Following enrollment, participants completed a baseline assessment (T0) using paper-and-pencil self-report questionnaires before the first program session in the presence of the researchers. The assessment included validated measures of integrative vitality, depression, fatigue, subhealth, subjective well-being, and mindfulness. Identifying information was collected on the questionnaires to link participants’ responses across assessments. Participants then took part in the 8-week MVP program. The program was delivered separately to three groups (Group A, *n* = 14; Group B, *n* = 12; and Group C, *n* = 9). All three groups were facilitated by the first author, a trained health psychologist and meditation instructor, with the second and third authors serving as co-facilitators and assisting with program delivery and attendance monitoring.

Following the final program session, participants completed a post-intervention assessment (T1) under the same administration procedures as at T0. The T1 assessment included the same measures administered at T0 and an additional program satisfaction survey. Participants who were subsequently classified as program dropouts were not separately invited to complete the T1 assessment. All program completers were invited in person at the final program session to participate in the qualitative component of the study, and semi-structured interviews were conducted within 1 month after program completion with those who voluntarily agreed to participate. Reasons for non-participation in the interviews were not collected. Participants classified as program dropouts were not interviewed.

The first author conducted all semi-structured interviews. At the time of the study, the first author was a research professor at a university in Seoul, Republic of Korea. He is male, holds a PhD in psychology, and is certified as both a health psychology specialist and a meditation instructor. He has experience in meditation instruction and qualitative research. As the first author also facilitated the MVP program, a researcher–participant relationship had been established during the intervention period prior to the interviews. Each participant was interviewed once for approximately 30 min in the researcher’s office, with only the participant and the interviewer present. All interviews were audio-recorded with participants’ consent. The interviews were conducted and transcribed verbatim in Korean, and qualitative analysis was conducted using the Korean transcripts. After the analysis, selected quotations were translated into English with the assistance of ChatGPT (OpenAI), and the researchers reviewed the translations against the original Korean quotations and revised them as necessary. No back-translation was performed. The semi-structured interview questions are presented in Table 1. The interview guide was not pilot-tested before data collection.

**Table 1:** Semi-structured interview questions.

	Items
1. Participant Self-Introduction	
2. Motivation and Expectations for Program Participation	
2–1	What did you hope to learn through the program?
2–2	Was there anything you wanted to change through the program?
3. Experiences During the Program	
3–1	What was the most memorable aspect of participating in the program?
3–2	What did you learn through the program?
3–3	What aspects of the program were satisfactory?
3–4	Were there any inconveniences or areas that need improvement?
4. Reflections After Completing the Program	
4–1	What changes have you experienced before and after the program?
4–2	Were you able to achieve what you wanted to learn or change through the program?

The first participant was enrolled on October 24, 2024, and the last participant was enrolled on February 5, 2025. Groups A and B participated in the MVP program from October 28 to December 16, 2024, whereas Group C participated from February 5 to March 26, 2025. Data collection was completed on April 3, 2025. Participants received KRW 100,000 as compensation for study participation, with an additional KRW 50,000 provided to those who participated in the semi-structured interview. The study flow is presented in Figure 1.

**Figure 1:**
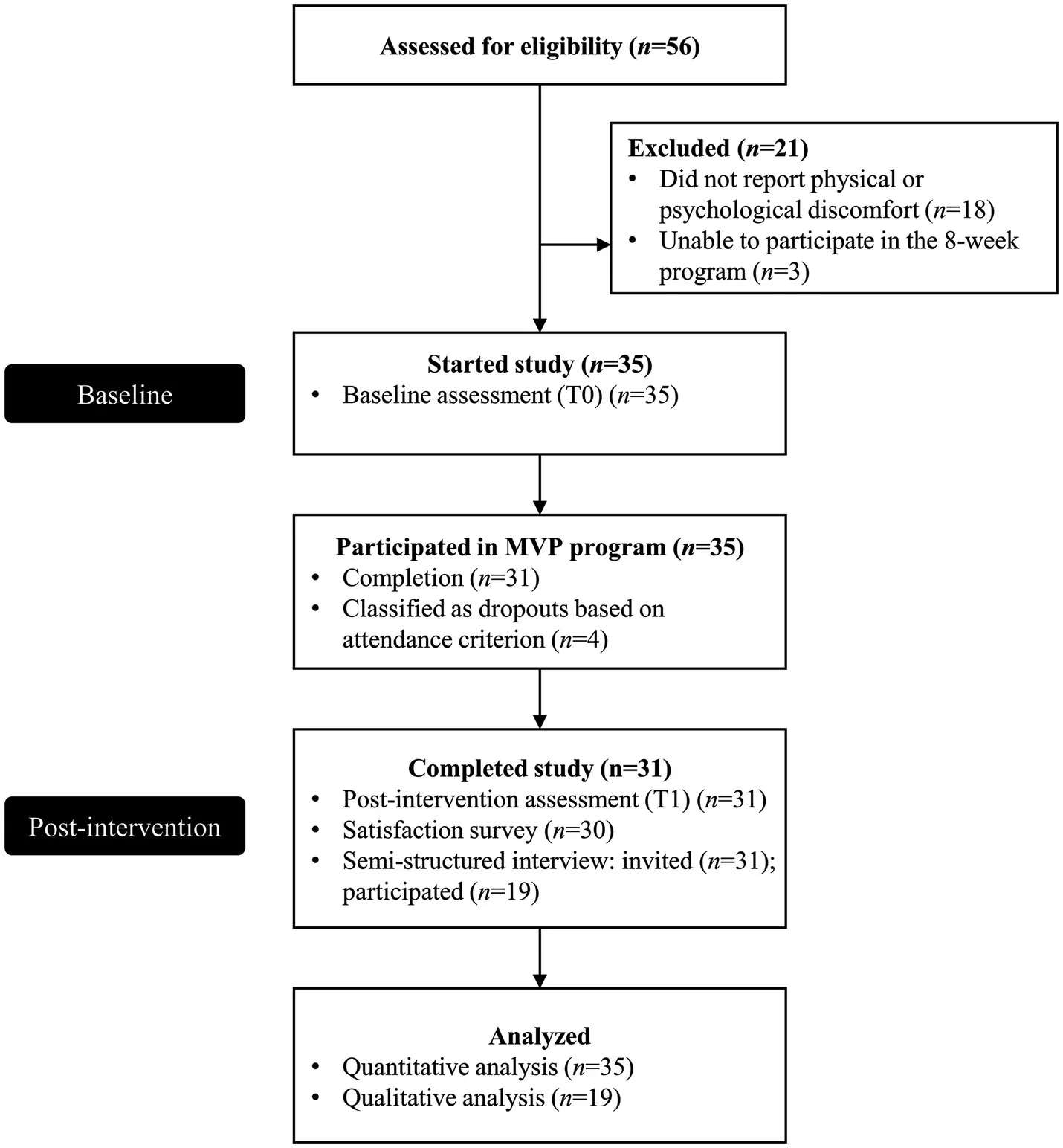
Study flow chart. MVP, Mindfulness-based vitality promotion.

### 2.4 MVP

MVP is a manualized program designed to enhance physical and psychological vitality. The content and procedures for each session were specified in an internally developed program manual, which was used consistently across all three groups. The program manual is available from the corresponding author upon reasonable request. The MVP consists of two modules: (i) relaxation and rest, and (ii) flow and activity. Yoon et al. (20) operationally defined physical vitality as a positive physical experience in a fully relaxed state and psychological vitality as a state of intrinsic motivation, interest, enjoyment in life, and active engagement. Accordingly, these two modules were designed to cultivate integrative vitality encompassing both physical and psychological aspects.

MVP suggests that balance and harmony are achieved when relaxation and rest alternate with flow and activity, leading to integrative vitality (20). Balance and harmony are key to maintaining optimal physical and psychological states. Relaxation response is a physiological homeostatic mechanism that restores an overly tense body to a healthy state (36). According to the general adaptation syndrome, physiological resources are depleted and exhausted under persistent chronic stress (37). Therefore, appropriate relaxation is essential for maintaining physiological homeostasis.

The relaxation and rest module consists of sessions 1–4. This module emphasizes breathing control, sufficient and high-quality sleep, a balanced diet, and mindfulness to achieve optimal rest. In the first session, participants were introduced to the principles of inhalation and exhalation and learned techniques to induce relaxation through controlled breathing. They then practiced breathing meditation. In the second session, participants learned about the protective and risk factors related to sleep and identified their own sleep routines. They practiced mindful yoga and performed a body scan. In the third session, participants explored the fundamental role of eating in energy intake and learned how to adopt balanced eating habits. Participants also practiced mindful eating. In the fourth session, participants learned about the relationship between the default mode network and distraction, as well as the principles of mindfulness, to help fully rest the body and mind. The participants engaged in mindfulness meditation by focusing on their awareness of breathing and bodily sensations.

The energy accumulated during relaxation and rest must be used meaningfully. Subsequently, MVP emphasizes sustainable flow and activity based on intrinsic motivation, which is distinct from pathological hyperarousal, such as the heightened energy and activity observed in hypomania (38). By contrast, the flow and activity module in MVP emphasizes autonomy, competence, and relatedness to foster intrinsic rather than extrinsic motivation.

The flow and activity module consists of sessions 5–8. In the fifth session, participants autonomously reflected on the meaning of happiness. They explored the happiness they genuinely desired, rather than externally imposed ideals, and engaged in walking meditation. In the sixth session, participants explored their personal values, developed step-by-step plans to achieve them, and gained a sense of competence through related practices. Activities included writing an epitaph and identifying role models. The participants also practiced breathing meditation by reciting phrases that reflected their personal values while focusing on their breaths. In the seventh session, participants learned about the concept of compassion and explored ways to incorporate compassionate practices into their daily lives. They then practiced compassion meditation. In the eighth session, participants developed plans to implement sustainable practices in their routines. They returned to their beginner’s minds and re-engaged in breathing meditation, which was introduced in the first session.

Table 2 presents the topics and contents covered in each program session. The participants engaged in MVP once a week for 8 weeks. Each session consisted of 20 min of education, 40 min of practice, and 30 min of group activities, including home practice review and experience sharing. MVP was conducted in a group format, with a maximum of 15 participants per group. The participants were encouraged to practice at home at least once a day. Audio files were provided to support home practice.

**Table 2:** Sessions of Mindfulness-based Vitality Promotion.

Module	Session	Subject	Education (20 min)	Practice (40 min)	Group activities (30 min)
Relaxation and Rest	1	Breathing	Understanding breathing; How to control breathing	Breathing meditation	Home practice review; Experience sharing; Question and answer
	2	Sleep	Understanding the protective and risk factors of sleep	Mindful yoga; Body scan	
	3	Eating	Understanding eating; How to eat in a balanced way	Mindful eating	
	4	Mindfulness	Understanding the default mode network Understanding mindfulness	Mindfulness meditation (breathing and bodily sensations)	
Flow and Activity	5	Happiness	Finding authentic happiness	Walking meditation	Home practice review; Experience sharing; Question and answer
	6	Values	Exploring my values; Achieving value in life	Breathing meditation (reciting personal values)	
	7	Connectedness	Understanding compassion; Trying compassion in life	Compassion meditation	
	8	Repeating	Making a sustainable practice plan	Breathing meditation	

### 2.5 Measurements

#### 2.5.1 Integrative Vitality Scale

The Integrative Vitality Scale (IVS), developed by members of the present research team, was used to assess physical and psychological vitality (20). The IVS was registered as the primary outcome measure, while the other outcome measures were registered as secondary outcomes. This scale is a 5-point Likert self-report scale consisting of 11 items for physical vitality (IVS-P) and 11 items for psychological vitality (IVS-E). The total score ranges from 0 to 44 for each subscale and from 0 to 88 for the overall IVS. A high score indicates a high level of physical or psychological vitality. In a previous study, the internal consistency was 0.91 for IVS-P, 0.91 for IVS-E, and 0.94 overall (20). In this study, the internal consistency was 0.91 for IVS-P, 0.95 for IVS-E, and 0.95 overall.

#### 2.5.2 Beck Depression Inventory

The Korean version of the Beck Depression Inventory (BDI) was used to assess symptoms of depression (39). It is a 4-point self-report scale consisting of 21 items. The total score ranges from 0 to 63. A high score indicates an increased level of depressive symptoms. The internal consistency was 0.85 in a previous study (39). In this study, the internal consistency was 0.89.

#### 2.5.3 Fatigue Scale

The Fatigue Scale (FS) was used to assess the fatigue levels (40). It is a 4-point Likert self-report scale consisting of 14 items. The total score ranges from 14 to 56. A high score indicates a high level of fatigue. In a previous study, the internal consistency was 0.84 (41). In this study, the internal consistency was 0.89.

#### 2.5.4 Mibyeong Index

The Mibyeong Index (MBI) was used to assess the level of Mibyeong (subhealth) (42). Mibyeong refers to a condition that is not diagnosed as a specific disease but presents with clear abnormal symptoms in the body, a pre-disease state (42). It is a 7-point Likert self-report scale consisting of 21 items. This scale encompasses four physical symptom subfactors (fatigue, pain, sleep disorders, and digestive issues) and three psychological symptom subfactors (depression, anger, and anxiety). Each subfactor evaluates symptom severity, duration, and degree of recovery after rest. The total score for each subfactor ranges from 3 to 21. A higher score indicates a greater degree of subhealth and more severe physical and psychological symptoms (42). In a previous study, the internal consistency was 0.91 overall (42). In this study, the internal consistency was 0.71 for fatigue, 0.89 for pain, 0.82 for sleep disorders, 0.91 for digestive issues, 0.90 for depression, 0.79 for anger, and 0.90 for anxiety.

#### 2.5.5 Concise Measure of Subjective Well-Being

The Concise Measure of Subjective Well-Being (COMOSWB) was used to assess subjective well-being (43). It is a 7-point Likert self-report scale consisting of nine items. This scale consists of three items, each on life satisfaction, positive affect, and negative affect. The total score was calculated by deducting the total score for negative affect from the sum of the life satisfaction and positive affect scores, with possible scores ranging from −15 to 39. A higher score indicates greater subjective well-being (43). In a previous study, the internal consistency was 0.818 for life satisfaction, 0.893 for positive affect, and 0.859 for negative affect (43). In this study, the internal consistency was 0.88 for life satisfaction, 0.81 for positive affect, and 0.80 for negative affect.

#### 2.5.6 Cognitive and Affective Mindfulness Scale-Revised

The Korean version of the Cognitive and Affective Mindfulness Scale-Revised (CAMS-R) was used to determine the level of mindfulness (44). It is a 4-point Likert self-report scale consisting of 10 items. The total score ranges from 10 to 40. A higher score indicates a greater level of mindfulness. The internal consistency was reported as 0.70 in a previous study (44). In this study, the internal consistency was 0.89.

#### 2.5.7 Satisfaction survey

A satisfaction survey was conducted to assess participants’ perceived satisfaction with the MVP program. The survey included six items rated on a 5-point Likert scale (0–4) and three open-ended questions (Supplementary material 2). It measured overall satisfaction and satisfaction with specific aspects of the program, such as its content, setting, and duration. Higher scores indicated greater satisfaction. For items assessing willingness to participate in the program again and willingness to recommend the program to others, responses of 3 (“agree”) or 4 (“strongly agree”) were considered positive responses. The participants were also asked to describe in detail the perceived strengths and weaknesses of the program in the open-ended questions.

### 2.6 Feasibility and acceptability outcomes

Feasibility and acceptability were evaluated descriptively based on program retention, session attendance, and participant satisfaction. Program retention was defined as completion of the 8-week program without meeting the predefined dropout criteria. Session attendance was assessed based on the number of sessions attended out of eight. Session-specific attendance rates were calculated using all enrolled participants (*n* = 35) as the denominator. Acceptability was assessed using the post-program satisfaction survey, including overall satisfaction, satisfaction with specific program components, willingness to participate again, and willingness to recommend the program to others.

No *a priori* progression criteria or quantitative thresholds for determining feasibility or acceptability were specified. Although participants were encouraged to practice mindfulness at home, home-practice adherence was not systematically recorded.

### 2.7 Data analyses

#### 2.7.1 Quantitative analysis

Statistical analyses were performed using SPSS version 22.0. The primary quantitative analyses were conducted using a full analysis set including all 35 enrolled participants. The individual participant was the unit of analysis. Four participants who were subsequently classified as program dropouts did not complete the T1 assessment. For these participants, T0 values were first carried forward to T1 using the last observation carried forward method. Subsequently, sporadic item-level missing responses within otherwise completed questionnaires were imputed using the expectation–maximization (EM) algorithm. At T0, EM imputation was applied to 21 missing item responses (IVS, 1; MBI, 17; COMOSWB, 3), and at T1, it was applied to nine missing item responses (IVS, 1; FS, 1; MBI, 7). One participant did not provide any COMOSWB responses at either T0 or T1 and was therefore excluded from analyses of this outcome (*n* = 34).

A frequency analysis was used to describe the participants’ sociodemographic characteristics. Paired *t*-tests were used to evaluate pre–post changes in the study outcomes. For paired *t*-tests, effect sizes were calculated as Cohen’s *d*_z_ using the mean change divided by the standard deviation of the change scores. Absolute values of *d*_z_ are reported to indicate the magnitude of the effect. The normality of the difference scores was assessed using the Shapiro–Wilk test. Significant deviations from normality were observed for IVS-P, BDI, FS, MBI, MBI-sleep, MBI-digestion, MBI-depression, MBI-anxiety, and COMOSWB. Wilcoxon signed-rank tests were additionally conducted as sensitivity analyses. Complete-case analyses restricted to program completers were also conducted during revision as sensitivity analyses. These analyses used the same EM-imputed dataset after excluding the four program dropouts, without repeating the imputation procedure. A *p*-value of < 0.05 was considered statistically significant.

#### 2.7.2 Qualitative analysis

Qualitative analysis was conducted using reflexive thematic analysis. Thematic analysis is a theoretically flexible method that involves the identification, analysis, and description of patterns in textual data (45, 46). The analysis followed Braun and Clarke’s (45) six-phase guidelines. The analysis adopted a predominantly experiential orientation, seeking to understand participants’ experiences as expressed in their accounts. Coding was primarily semantic, while underlying meanings were also considered where relevant to theme development.

The first and second authors, who served as the program facilitator and co-facilitator, respectively, were involved in coding and theme development. Rather than seeking inter-coder agreement, the coders discussed their interpretations throughout the analytic process to support the development and refinement of themes. Differences in interpretation were considered through reflexive discussion rather than treated as coding discrepancies requiring resolution. No qualitative data analysis software was used.

As the program facilitator, the first author approached the interviews with a particular interest in understanding participants’ experiences of the MVP program and remained aware that his prior involvement with the program and participants could shape both the interview process and interpretation of the data. To enhance the trustworthiness of the analysis and promote reflexivity, regular meetings were held with the research team throughout the analytic process. During these meetings, the data, codes, and themes were reviewed from multiple perspectives, and alternative interpretations were critically considered. An inductive approach was used, with codes and themes derived primarily from the interview data rather than from a predetermined theoretical or coding framework.

#### 2.7.3 Mixed-methods integration

Quantitative and qualitative data were initially analyzed separately and subsequently integrated at the interpretation and reporting stage. Integration involved comparing findings across the two components to examine their fit, including areas of confirmation, expansion, and discordance, and to develop an integrated interpretation of the program outcomes (47). A joint display was used to present the major quantitative and qualitative findings and their integrated interpretations.

## 3 Results

### 3.1 Participant characteristics

Among 56 individuals who expressed interest in the program, 35 (62.5%) were enrolled in the study; 18 did not report physical or psychological discomfort and three were unable to participate in the 8-week program. The average age was 53.74 years, ranging from 28 to 79 years. Most participants (71.4%) had at least a four-year university degree, and homemakers constituted the largest occupational group (40.0%). Based on the sex-specific K-BDI severity criteria (39), 17 participants (48.6%) were classified as normal, 11 (31.4%) as having mild depression, 3 (8.6%) as having moderate depression, and 4 (11.4%) as having severe depression at baseline. Table 3 presents the demographic characteristics of the participants.

**Table 3:** Participants’ demographic characteristics and feasibility and acceptability outcomes.

Variable	Mean ± SD or Rate% (*n*)
Sex	
Male	8.6 (3)
Female	91.4 (32)
Age	53.74 ± 12.45
20s	2.9 (1)
30s	8.6 (3)
40s	28.6 (10)
50s	22.9 (8)
60s and above	37.1 (13)
Level of education	
High School Graduate	17.1 (6)
Graduated from a Two-Year College	11.4 (4)
Graduated from a Four-Year University	51.4 (18)
Enrolled in / Completed Master’s Coursework	5.7 (2)
Master’s Degree	5.7 (2)
Enrolled in / Completed Doctoral Coursework	2.9 (1)
Doctoral Degree	5.7 (2)
Occupation	
Clerical / Administrative and Managerial Occupations	11.4 (4)
Professional Occupations	14.3 (5)
Service and Sales Occupations	2.9 (1)
Homemaker	40.0 (14)
Unemployed	22.9 (8)
Other	8.6 (3)
Feasibility	
Program retention	88.6 (31)
Sessions attended	6.37 ± 1.78
Session-specific attendance	
1st Session	82.9 (29)
2^nd^ Session	85.7 (30)
3rd Session	80.0 (28)
4th Session	82.9 (29)
5th Session	68.6 (24)
6th Session	82.9 (29)
7th Session	68.6 (24)
8th Session	85.7 (30)
Acceptability	
Overall satisfaction	3.70 ± 0.54
Content	3.80 ± 0.41
Setting	3.70 ± 0.65
Duration	3.60 ± 0.67
Willingness to participate again	3.73 ± 0.58
Positive response^a^ (n/respondents)	93.3 (28/30)
Positive response^a^ (n/enrolled)	80.0 (28/35)
Willingness to recommend to others	3.83 ± 0.38
Positive response^a^ (n/respondents)	100.0 (30/30)
Positive response^a^ (n/enrolled)	85.7 (30/35)

a Positive responses were defined as scores of 3 (“agree”) or 4 (“strongly agree”). Satisfaction survey data were available for 30 participants; the five participants without these data were included in the enrolled-sample denominator but not counted as positive responses.

### 3.2 Feasibility and acceptability

Table 3 presents participants’ retention, session attendance, and satisfaction with the MVP program. Of the 35 enrolled participants, 31 completed the program, corresponding to a retention rate of 88.6%, whereas four participants (11.4%; all female) were classified as dropouts based on the predefined attendance criterion. Nonattendance among these participants began in week 2 for one participant, week 3 for two participants, and week 5 for one participant. The reasons for nonattendance were not ascertained. Across all enrolled participants, the mean number of sessions attended was 6.37 out of eight. Among the 31 program completers, 10 attended all eight sessions, 10 attended seven sessions, 9 attended six sessions, and 2 attended five sessions. Session-specific attendance rates, calculated using all enrolled participants (*n* = 35) as the denominator, ranged from 68.6 to 85.7% (Table 3).

Thirty of the 31 program completers completed the satisfaction survey; one participant did not return the survey. The mean overall satisfaction score was 3.70, with mean scores of 3.80 for program content, 3.70 for setting, and 3.60 for duration. Among the 30 respondents, 28 (93.3%) provided a positive response regarding willingness to participate in the program again, and all 30 (100%) provided a positive response regarding willingness to recommend the program to others. Open-ended responses highlighted positive experiences with meditation and breathing practices, particularly for self-reflection, emotional well-being, and application in daily life. Suggestions for improvement mainly concerned the program schedule and duration, while some participants expressed interest in continued participation.

### 3.3 Pre–post changes in quantitative outcomes

Table 4 presents the paired *t*-test results for the outcomes. The IVS, IVS-P, IVS-E, and COMOSWB scores were significantly higher at T1 than at T0. In contrast, the BDI, FS, MBI, MBI-fatigue, MBI-depression, and MBI-anxiety scores were significantly lower at T1 than at T0. However, no significant differences were observed between T0 and T1 for CAMS-R, MBI-pain, MBI-sleep, MBI-digestion, and MBI-anger.

**Table 4:** Changes in study outcomes from baseline to post-intervention.

Variable	Baseline (T0)	Post-intervention (T1)	Mean change ± SD [95% CI]	*t*	*p*	*|d*_z_ *|*
IVS	48.57 ± 14.64	59.40 ± 15.47	10.83 ± 11.37 [6.92, 14.74]	5.63	<0.001	0.95
IVS-P	22.00 ± 7.38	28.69 ± 8.11	6.69 ± 6.19 [4.56, 8.81]	6.39	<0.001	1.08
IVS-E	26.57 ± 8.52	30.71 ± 8.43	4.14 ± 6.67 [1.85, 6.44]	3.67	<0.001	0.62
BDI	15.66 ± 7.62	11.11 ± 8.30	−4.54 ± 5.47 [−6.42, −2.66]	−4.91	<0.001	0.83
FS	33.69 ± 7.15	28.64 ± 7.06	−5.05 ± 6.09 [−7.14, −2.96]	−4.90	<0.001	0.83
MBI	64.26 ± 22.28	53.45 ± 20.99	−10.81 ± 20.22 [−17.76, −3.87]	−3.16	0.003	0.53
MBI-fatigue	10.07 ± 3.68	8.37 ± 3.12	−1.70 ± 3.96 [−3.06, −0.34]	−2.54	0.016	0.43
MBI-pain	9.92 ± 4.46	8.17 ± 3.82	−1.74 ± 5.20 [−3.53, 0.04]	−1.98	0.055	0.33
MBI-sleep	10.09 ± 4.12	8.60 ± 4.26	−1.49 ± 4.48 [−3.03, 0.05]	−1.97	0.057	0.33
MBI-digestion	7.49 ± 4.91	7.06 ± 4.72	−0.43 ± 3.19 [−1.52, 0.67]	−0.79	0.435	0.13
MBI-depression	9.12 ± 4.82	6.93 ± 4.34	−2.19 ± 4.06 [−3.58, −0.79]	−3.19	0.003	0.54
MBI-anger	7.95 ± 4.18	6.54 ± 3.40	−1.41 ± 4.15 [−2.84, 0.02]	−2.01	0.053	0.34
MBI-anxiety	9.63 ± 4.92	7.77 ± 4.27	−1.85 ± 3.70 [−3.12, −0.58]	−2.97	0.005	0.50
COMOSWB	15.02 ± 8.98	20.09 ± 10.89	5.07 ± 7.38 [2.50, 7.64]	4.01	<0.001	0.69
CAMS-R	27.14 ± 6.03	28.20 ± 5.73	1.06 ± 4.33 [−0.43, 2.55]	1.44	0.158	0.24

IVS, Integrative Vitality Scale; IVS-P, physical vitality subscale; IVS-E, psychological vitality subscale; BDI, Korean version of the Beck Depression Inventory; FS, Fatigue Scale; MBI, Mibyeong Index; COMOSWB, Concise Measure of Subjective Well-Being; CAMS-R, Korean version of the Cognitive and Affective Mindfulness Scale-Revised. Mean change was calculated as T1 − T0. Analyses included 35 participants for all outcomes except COMOSWB (*n* = 34).

Sensitivity analyses using Wilcoxon signed-rank tests yielded generally consistent results with the paired *t*-test. MBI-pain and MBI-sleep were the only outcomes for which statistical significance differed between the two approaches. Full results are provided in Supplementary material 3. The complete-case analyses yielded the same pattern of statistical significance as the primary analyses (Supplementary material 4).

### 3.4 Thematic analysis

Semi-structured interviews were conducted with 19 voluntary participants (16 females) of the 31 individuals who completed the MVP program. Table 5 presents the sociodemographic characteristics of the participants. Reflexive thematic analysis yielded a total of four themes and 13 subthemes (Table 6). The four themes were: (1) diverse life challenges and self-care needs, (2) learning through experience, connection, and application, (3) regaining balance and agency in life, and (4) sustaining practice requires continued support.

**Table 5:** Demographic characteristics of semi-structured interview participants.

Participant no.	Age	Sex	Level of education	Occupation
P1	≥60	Female	Graduated from a Two-Year College	Homemaker
P2	40–49	Female	Graduated from a Four-Year University	Clerical / Administrative and Managerial Occupations
P3	50–59	Female	Graduated from a Four-Year University	Homemaker
P4	≥60	Female	High School Graduate	Homemaker
P5	≥60	Male	Graduated from a Four-Year University	Unemployed
P6	40–49	Female	Graduated from a Four-Year University	Other
P7	50–59	Female	Enrolled in / Completed Doctoral Coursework	Other
P8	≥60	Female	High School Graduate	Homemaker
P9	≥60	Male	Enrolled in / Completed Master’s Coursework	Unemployed
P10	40–49	Female	Master’s Degree	Professional Occupations
P11	≥60	Female	High School Graduate	Homemaker
P12	50–59	Female	Graduated from a Four-Year University	Homemaker
P13	≥60	Female	Graduated from a Four-Year University	Homemaker
P14	≥60	Male	Doctoral Degree	Other
P15	40–49	Female	Graduated from a Four-Year University	Unemployed
P16	50–59	Female	Doctoral Degree	Professional Occupations
P17	40–49	Female	Graduated from a Four-Year University	Professional Occupations
P18	40–49	Female	Graduated from a Four-Year University	Professional Occupations
P19	40–49	Female	Graduated from a Four-Year University	Homemaker

**Table 6:** Summary of themes and examples from semi-structured interviews.

Themes	Sub-themes	Examples from participant interviews
Diverse life challenges and self-care needs	Navigating age- and life-related transitions	“As I’m getting closer to 60—though I still have some time—I find myself wondering, ‘How should I move forward? How am I going to get through this?’ I sometimes get caught up in thoughts like these and feel a sense of urgency.” (P16)
	Coping with emotional and everyday stress	“I worked for about 35 or 36 years, and when I was working, I was always so busy that I did not really have much time to think about these things. Now that I have more time, I sometimes feel a little more isolated than I used to, and I’ve noticed that my emotions have changed as well.” (P5)
	Living with diminished vitality	“So yeah, ‘vitality’ really hits home for me these days. I’ve been super tired and just kinda low-energy, and I really want to feel more alive, you know? Even a friend I had not seen in a while told me I seemed like I was getting weak or worn out. I guess I’ve just been feeling a bit sluggish in everyday life.” (P15)
Learning through experience, connection, and application	Engagement in new experiences and learning	“You know, we are always breathing as we go about our lives, but we do not usually notice our breathing, right? But on that first day, I became really aware of my breath—and it kind of surprised me. Like, ‘Wait, is my breathing really this irregular? Have I been breathing this badly all this time?’ It made me realize just how unaware I’d been of all that.” (P16)
	Interpersonal interaction within the program	“One of the participants said something that really stuck with me. She was like, ‘All my life, I’ve just been living for my husband and kids. That’s all I ever did. But over time, I realized—that’s not it. I need to think about myself first. Then I can care for my kids and the people around me. That’s actually the wiser way to live.’ The way she said that really hit home for me.” (P8)
	Acquisition of life-applicable skills	“It has not been some big transformation, but it’s helped me make things feel a bit more manageable and comfortable. It kind of gave me a chance to make my life a little easier.” (P6)
Regaining balance and agency in life	Somatic relaxation	“And honestly, I used to get really bad headaches. But the thing is, I could not even tell if it was a headache, or if I was just sleepy, or totally worn out. So I kept trying to fix it by taking naps during the day. But now, that’s gone.” (P10)
	Emotional stability	“Whenever those dark thoughts come up, I try to meditate and take a few deep breaths — like really deep in and out. And it actually helps. It calms me down a lot. That’s been really helpful for me.” (P1)
	Self-efficacy	“Like… I started to feel like I could actually manage myself better, not just emotionally, but even physically. You know how sometimes your body feels off, and you are like, ‘Should I go to the doctor? But even if I do, will they really be able to fix this?’ It’s not like you do not know what’s going on, but you just do not know what to do about it. But now, I feel like I’ve learned how to handle those things a bit better. Like I can manage it instead of just feeling stuck.” (P10)
	Positive relationship-building	“I’m thankful that I’m alive, and that gratitude really means a lot to me… Thinking about being thankful, like even when I meet neighbors today, I just naturally greet them more brightly and warmly. There’s something like that. So, even though I have a lot of worries and the situation is not that great, I’m still thankful just for being alive.” (P15)
	Authentic life navigators	“Now the kids are starting to be able to do things on their own more and more. So I’ll have more free time too, and I still probably have about half my life left to live, you know, as much as I’ve already lived. So now I want to find something I’m good at and something I really enjoy doing.” (P19)
Sustaining practice requires continued support	Challenges in maintaining regular practice	“I’m just busy living my life, you know. …Sometimes I forget, and then when I lie down at night, I’m like, ‘Oh, yeah.’ …Getting ready for work, making food, and all that — I just end up forgetting.” (P3)
	Need for follow-up support	“I take a lot of lifelong learning classes. But one thing I find a bit frustrating is that they do not really continue. Like, in a regular school, you have grades — first grade, second grade, third grade — and then you move on to high school, then college, so it’s a continuous process. But here, you just take one class, and there’s no follow-up or next level after that.” (P14)

#### 3.4.1 Diverse life challenges and self-care needs

Participants described diverse physical, emotional, and life-related challenges that contributed to a perceived need for self-care. Although their individual circumstances varied, their accounts reflected difficulties associated with aging and life transitions, emotional and everyday stress, and diminished vitality. These diverse experiences formed the context in which participants engaged with the MVP program.

##### 3.4.1.1 Navigating age- and life-related transitions

Participants described difficulties associated with aging and major life transitions. Some expressed uncertainty and a sense of urgency as they became more aware of growing older, while others experienced psychological or physical changes associated with aging. These experiences prompted participants to consider how they might better care for their physical and psychological well-being. *“As I’m getting closer to 60—though I still have some time—I find myself wondering, ‘How should I move forward? How am I going to get through this?’ I sometimes get caught up in thoughts like these and feel a sense of urgency.” (P16).*

##### 3.4.1.2 Coping with emotional and everyday stress

Participants also described emotional and everyday difficulties arising from different life circumstances. These included feelings of isolation and emotional changes after leaving long-term employment, stress experienced during the COVID-19 pandemic, and irritability associated with parenting. *“I worked for about 35 or 36 years, and when I was working, I was always so busy that I did not really have much time to think about these things. Now that I have more time, I sometimes feel a little more isolated than I used to, and I’ve noticed that my emotions have changed as well.” (P5)* Despite the different sources of stress, participants expressed a need to better manage their emotional experiences and care for their psychological well-being.

##### 3.4.1.3 Living with diminished vitality

Participants also described experiences of diminished vitality, including fatigue, low energy, physical weakness, and health problems that interfered with everyday functioning. Some became more aware of declining physical capacity following illness or with increasing age, while others described a general sense of lethargy and reduced energy in daily life. These experiences heightened their awareness of the need to restore vitality and better care for their health. *“So yeah, ‘vitality’ really hits home for me these days. I’ve been super tired and just kinda low-energy, and I really want to feel more alive, you know? Even a friend I hadn’t seen in a while told me I seemed like I was getting weak or worn out. I guess I’ve just been feeling a bit sluggish in everyday life.” (P15).*

#### 3.4.2 Learning through experience, connection, and application

Participants described gaining experiential knowledge through mindfulness practices and new insights through interpersonal interactions. They reported applying mindfulness techniques to address personal life challenges.

##### 3.4.2.1 Engagement in new experiences and learning

Rather than delivering conceptual knowledge, MVP emphasizes experiential learning. Through active engagement in various mindfulness practices and group activities, participants became aware of aspects of their lives that had previously gone unnoticed and developed their own experiential wisdom. For example, they learned how to be mindful of the present moment and became more familiar with their bodies. *“You know, we are always breathing as we go about our lives, but we do not usually notice our breathing, right? But on that first day, I became really aware of my breath—and it kind of surprised me. Like, ‘Wait, is my breathing really this irregular? Have I been breathing this badly all this time?’ It made me realize just how unaware I’d been of all that.” (P16)* Furthermore, they reflected on their past and began planning for the future. *“Usually I’m just focused on getting through the day or the week, but this time I actually tried to look a bit further ahead—like, ‘What do I really want? Where do I want to end up?’ It got me thinking more seriously about the future.” (P19).*

##### 3.4.2.2 Interpersonal interaction within the program

Participants described interpersonal interactions within the program as an important part of their learning experience. Conducted in small groups of approximately 10 participants, the program includes group sharing of experiences and practices. This setting allowed the participants to articulate their thoughts, engage with others, and learn from one another. *“One of the participants said something that really stuck with me. She was like, ‘All my life, I’ve just been living for my husband and kids. That’s all I ever did. But over time, I realized—that’s not it. I need to think about myself first. Then I can care for my kids and the people around me. That’s actually the wiser way to live.’ The way she said that really hit home for me.” (P8).*

##### 3.4.2.3 Acquisition of life-applicable skills

MVP teaches mindfulness techniques that can be applied to manage everyday challenges. Although participants noted that the program did not radically transform their daily habits, many reported that they were able to apply what they had learned in specific contexts, particularly during stressful events. *“It has not been some big transformation, but it’s helped me make things feel a bit more manageable and comfortable. It kind of gave me a chance to make my life a little easier.” (P6).*

#### 3.4.3 Regaining balance and agency in life

Participants described perceived changes in physical and psychological well-being, including somatic relaxation, emotional stability, increased self-efficacy, more positive interpersonal relationships, and a renewed sense of agency in their lives.

##### 3.4.3.1 Somatic relaxation

MVP emphasizes mind–body relaxation through sufficient rest. Participants reported improvements in stress-related physical symptoms, including headaches, sleep disturbances, and digestive issues, as well as an increased ability to manage physical discomfort. *“And honestly, I used to get really bad headaches. But the thing is, I could not even tell if it was a headache, or if I was just sleepy, or totally worn out. So I kept trying to fix it by taking naps during the day. But now, that’s gone.” (P10) “During those two months of meditation, I hardly felt it [the headache]—maybe only once or so—and I do not think it caused much discomfort. And whenever I did feel that discomfort, if I just took about three deep breaths, it felt like the discomfort was somewhat delayed.” (P10) “While doing practices like the body scan, I gradually began to feel calmer. At home, when I could not fall asleep late at night, I would listen to the recording the instructor had sent us. As I listened, I would feel myself relaxing, and before I knew it, I would start to drift off to sleep.” (P8) “When I feel a sense of urgency and my stress builds up, my bowel movements tend not to be very good. But when I feel more settled and at ease, they tend to be more like my usual bowel movements. Looking at it that way, I feel like it has become somewhat more manageable.” (P12).*

##### 3.4.3.2 Emotional stability

Participants described experiencing greater emotional calmness during and after mindfulness practice. Participants noted that they were better able to maintain emotional balance and avoid rumination when facing anxiety or depressive moods. *“Whenever those dark thoughts come up, I try to meditate and take a few deep breaths — like really deep in and out. And it actually helps. It calms me down a lot. That’s been really helpful for me.” (P1).*

##### 3.4.3.3 Self-efficacy

As participants effectively managed their physical and emotional difficulties, they developed a greater sense of control over their bodies and minds. For example, one participant described gaining confidence in managing digestive issues through meditation. *“Like… I started to feel like I could actually manage myself better, not just emotionally, but even physically. You know how sometimes your body feels off, and you are like, ‘Should I go to the doctor? But even if I do, will they really be able to fix this?’ It’s not like you do not know what’s going on, but you just do not know what to do about it. But now, I feel like I’ve learned how to handle those things a bit better. Like I can manage it instead of just feeling stuck.” (P10)* Moreover, participants described greater self-acceptance. *“Getting older, not being ‘productive’ anymore… I started to wonder if I was becoming someone the world did not really need. But lately, I’ve started to feel like… maybe it’s okay. Maybe it’s okay to live like this, to take my time, learn, figure things out as I go.” (P3).*

##### 3.4.3.4 Positive relationship-building

Participants described more positive interpersonal relationships following the MVP program. Participants expressed compassion not only toward close relations (e.g., spouses) but also toward strangers (e.g., people they passed on the street) and even expressed gratitude for life itself. *“I’ve come to understand my husband, who is the person closest to me, much better. As I’ve become more at ease myself, I naturally speak to him more gently. I used to point out things he did quite often, but now I’m more likely to think, ‘Well, I can see why he might do that,’ and I do not criticize him as much. As a result, we hardly have any conflicts these days.” (P8) “I’m thankful that I’m alive, and that gratitude really means a lot to me… Thinking about being thankful, like even when I meet neighbors today, I just naturally greet them more brightly and warmly. There’s something like that. So, even though I have a lot of worries and the situation is not that great, I’m still thankful just for being alive.” (P15).*

##### 3.4.3.5 Authentic life navigators

Beyond relaxation, MVP provided opportunities for self-reflection aimed at living a more flourishing life. The participants discovered meaningful values and aspirations and decided to take ownership of their lives. *“Now the kids are starting to be able to do things on their own more and more. So I’ll have more free time too, and I still probably have about half my life left to live, you know, as much as I’ve already lived. So now I want to find something I’m good at and something I really enjoy doing.” (P19).*

#### 3.4.4 Sustaining practice requires continued support

Participants described difficulties maintaining regular mindfulness practice after the program ended and expressed a need for continued support.

##### 3.4.4.1 Challenges in maintaining regular practice

The participants experienced difficulty continuing their mindfulness practice after the program ended. Although they recognized positive changes in practice, these experiences did not always lead to sustained practice. *“I’m just busy living my life, you know. …Sometimes I forget, and then when I lie down at night, I’m like, ‘Oh, yeah.’ …Getting ready for work, making food, and all that — I just end up forgetting.” (P3)* Barriers such as demanding work schedules, caregiving responsibilities, and the absence of a facilitator made it challenging to maintain regular practice.

##### 3.4.4.2 Need for follow-up support

One participant described difficulties maintaining a calm state after discontinuing regular meditation practice. *“Now that it’s been a few days since I last meditated, I feel like my mind, which I had sort of calmed down, is starting to wander off in a different direction again.” (P8)* Participants also expressed a desire for follow-up support to sustain their practice. One participant criticized the one-off nature of the programs offered at lifelong learning centers and called for the development of sustainable educational systems. *“I take a lot of lifelong learning classes. But one thing I find a bit frustrating is that they do not really continue. Like, in a regular school, you have grades—first grade, second grade, third grade—and then you move on to high school, then college, so it’s a continuous process. But here, you just take one class, and there’s no follow-up or next level after that.” (P14)* Another participant suggested creating an online community to motivate daily mindfulness practice.

No clear accounts of symptom worsening or other unwanted effects associated with the MVP program were identified in the interview data.

### 3.5 Integration of quantitative and qualitative findings

Integration of the quantitative and qualitative findings revealed areas of expansion and differences in the information provided by the two components (Table 7). The qualitative findings provided additional experiential context for the quantitative changes in integrative vitality, subjective well-being, and physical and emotional symptoms. For pain, sleep disturbance, and anger, quantitative point estimates were in the direction of improvement but remained imprecise, while participants described perceived improvements in these areas. For digestive symptoms, the quantitative estimate was close to zero despite perceived improvements reported by some participants. Participants also described changes in their understanding and application of mindfulness, although the corresponding quantitative measure did not show a statistically significant change. In addition, the qualitative findings identified challenges in sustaining mindfulness practice after the program that were not quantitatively assessed.

**Table 7:** Joint display of mixed-methods findings.

Domain	Quantitative findings	Qualitative findings	Integrated interpretation
Integrative vitality	Integrative vitality significantly increased from baseline (T0) to post-intervention (T1). Physical and psychological vitality also significantly increased.	Participants described somatic relaxation and emotional stability, as well as a greater sense of agency and engagement with personally meaningful ways of living.	Expansion. Qualitative findings provided experiential context for the observed improvements in integrative vitality and its physical and psychological domains.
Subjective well-being	Subjective well-being significantly increased from T0 to T1.	Participants described more positive relationships with themselves and others and greater engagement with personally meaningful ways of living.	Expansion. Qualitative findings provided additional context for the observed improvement in subjective well-being by illustrating relational and personally meaningful aspects of participants’ experiences.
Fatigue, depression, and anxiety	Significant reductions were observed in fatigue, depression, and anxiety.	Participants described experiences of somatic relaxation and emotional stability following participation in the program.	Expansion. Qualitative findings provided experiential context for the observed reductions in physical and emotional symptoms.
Pain, sleep, digestive symptoms, and anger	No statistically significant changes were observed in pain, sleep disturbance, digestive symptoms, or anger. Point estimates for pain, sleep disturbance, and anger were in the direction of improvement but imprecise, whereas the estimate for digestive symptoms was close to zero.	Participants described an increased ability to manage physical discomfort, improvements in sleep and digestive symptoms, and better anger regulation accompanied by reduced interpersonal conflict.	Expansion. Qualitative findings provided additional context for imprecise quantitative estimates and described individual experiences not necessarily reflected in group-level changes. These findings do not establish improvements in these symptom domains.
Mindfulness	Self-reported mindfulness showed no statistically significant change.	Participants described increased awareness and acceptance and reported applying mindfulness principles in everyday life.	Discordance and expansion. Participants described changes in their understanding and application of mindfulness that were not reflected in self-reported mindfulness scores.
Feasibility and acceptability	Of 35 enrolled participants, 31 (88.6%) completed the program, with a mean attendance of 6.37 of eight sessions. Satisfaction among respondents was generally favorable. Post-program maintenance of mindfulness practice was not quantitatively assessed.	Participants valued experiential learning, interpersonal connection, and skills applicable to everyday life. They also described difficulties sustaining regular mindfulness practice after the program and expressed a need for continued support.	Expansion. Qualitative findings provided additional context for acceptability by identifying valued aspects of the program and highlighted challenges to sustained practice that were not captured by the quantitative measures.

## 4 Discussion

This study employed a mixed-methods design to evaluate the feasibility and acceptability of the MVP program and explore pre–post changes in health-related outcomes. The findings suggest that MVP may be suitable for implementation in community-based lifelong learning institutions, with exploratory findings indicating changes in physical and psychological health-related outcomes. This section discusses the main findings, limitations, and implications for future research.

The findings suggest that MVP may have the potential to promote holistic health among community-dwelling adults. Quantitative analyses revealed significant pre–post increases in integrative vitality and subjective well-being, along with significant reductions in fatigue, depression, and anxiety. These findings align with previous research suggesting that mindfulness training induces physiological relaxation responses (48), improves emotional regulation (49), and enhances well-being (50). Mindfulness-based programs have been investigated for clinical issues such as stress in chronic illness, depression, and relapse prevention in addiction (51–53) and have also been increasingly studied in nonclinical settings, including workplaces, schools, and communities (54–56). A systematic review and meta-analysis found that such programs may improve anxiety, depression, distress, and well-being relative to no intervention in nonclinical settings; however, effects varied across settings, and there was no clear evidence of superiority over other preventive interventions (57). Importantly, MVP is a multicomponent intervention that combines mindfulness practice with other behavioral and educational components, including relaxation, health-related education, and activities aimed at supporting meaningful engagement. Therefore, the pre–post changes observed in the present study cannot be attributed specifically to mindfulness practice or to any individual component of MVP.

The qualitative findings provided further context for understanding participants’ experiences with the MVP program. Participants entered the program with diverse self-care needs related to age- and life-related transitions, emotional and everyday stress, and diminished vitality. Despite this heterogeneity, participants described engaging with the program through experiential learning, interpersonal connection, and the application of mindfulness skills to everyday life. Participants also described somatic relaxation and emotional stability, as well as a greater sense of agency in their lives. They reported more positive relationships with themselves and others and greater engagement with personally meaningful goals. The Integrative Vitality Model, previously developed by members of the present research team (20), proposes that sustainable well-being may be supported by the restoration of physical vitality through relaxation and subsequent engagement in meaningful activities. The present qualitative findings were broadly compatible with this proposed pathway; however, the current study was not designed to test this mechanism. Future controlled studies are needed to directly examine the hypothesized pathway.

Differences in the information provided by the quantitative and qualitative findings warrant consideration (Table 7). First, the paired *t*-tests did not show statistically significant changes in pain, sleep disturbance, digestive symptoms, or anger. However, the point estimates for pain, sleep disturbance, and anger were in the direction of improvement, with 95% confidence intervals narrowly including zero, indicating uncertainty rather than clear discordance with the qualitative findings. The statistical significance of pain and sleep disturbance also varied depending on the statistical test used (Supplementary material 3). In contrast, the point estimate for digestive symptoms was close to zero. Participants described an increased ability to manage physical discomfort, improvements in sleep and digestive symptoms, and better anger regulation accompanied by reduced interpersonal conflict. These findings do not establish improvements in these symptom domains. The differences between individual experiences and group-level estimates may partly reflect the heterogeneity of participants’ baseline concerns, which was also evident in the qualitative findings. Participants entered the program with diverse physical, emotional, and life-related difficulties rather than a single shared problem. The inclusion criteria for this study were broad, allowing for the participation of adults experiencing physical or psychological distress regardless of specific diagnoses. Indeed, while some participants mainly reported emotional issues such as depression, anxiety, or feelings of alienation, others reported physical concerns such as pain, sleep disturbances, or digestive problems. Consequently, changes in specific symptom domains may have been relevant to only a subset of participants and may not have been fully reflected in group-level mean changes.

Second, the quantitative data showed no statistically significant improvements in mindfulness levels. Given that mindfulness is a key component of MVP, the absence of a significant change in self-reported mindfulness warrants consideration. In contrast, qualitative findings provided a different perspective. Participants demonstrated a clear understanding of mindfulness principles, such as awareness and acceptance, and reported actively applying them to their daily lives. This discrepancy may partly reflect limitations of self-reported measures in assessing mindfulness (58). Self-report questionnaires require introspection, awareness, and reflection on one’s own thoughts, feelings, attitudes, and behaviors. As mindfulness itself involves intentional and objective introspection, assessing mindfulness without being in a mindful state may lack validity. A previous study has shown that meditators and non-meditators respond differently to mindfulness scale items, indicating potential threats to the construct validity of pre–post assessments of mindfulness (58). Similarly, in the present study, one participant noted that after learning mindfulness, they realized how unaware they had previously been in daily life. *“I think meditation helped me realize that I wasn’t really staying in the moment here. …By practicing it over and over, I’m starting to get what it really means to just stay right here, right now.” (P18)* Another participant reported noticing an irregularity of breathing only after engaging in meditation. *“But on that first day, I became really aware of my breath—and it kind of surprised me. Like, ‘Wait, is my breathing really this irregular? Have I been breathing this badly all this time?’ It made me realize just how unaware I’d been of all that.” (P16)* These examples are consistent with the possibility that changes in self-awareness may affect how participants evaluate their own mindfulness. However, alternative explanations should be given equal consideration. The program may not have substantially changed the aspects of mindfulness assessed by the CAMS-R, or the CAMS-R may not have been sufficiently sensitive to detect changes in this population.

The findings provide preliminary information regarding the feasibility and acceptability of implementing MVP within a lifelong learning institution. Of the 35 enrolled participants, 31 (88.6%) completed the program, and participants attended an average of 6.37 of eight sessions. Program satisfaction among respondents was generally favorable. However, because no *a priori* progression criteria were established, these findings should be interpreted descriptively rather than as demonstrating that predetermined feasibility criteria were met. Qualitative findings provided additional context for acceptability, as participants valued experiential learning, interpersonal connection, and the acquisition of skills applicable to everyday life. Collectively, these findings can inform the design of future controlled studies with prospectively defined criteria for evaluating feasibility and acceptability.

Participants commonly described difficulties sustaining regular mindfulness practice after the program. Similarly, a previous qualitative study confirmed that incorporating mindfulness training into daily routines is challenging (59). In that study, the main barriers included unexpected stressful events, diminished motivation, and the absence of instructors. These findings suggest the need for follow-up support to help participants sustain mindfulness practices beyond the program duration. Based on the participants’ feedback, we propose the following follow-up strategies: First, advanced-level mindfulness programs should be developed for more experienced practitioners. Second, in addition to regular courses, non-continuous sessions, such as retreats, should be offered on a weekly or monthly basis. Third, online and offline platforms such as learning communities and social networking groups should be established to facilitate peer connections among program alumni. Successful implementation of these follow-up strategies requires active collaboration among lifelong learning institutions, program coordinators, and instructors.

### 4.1 Limitations

This study has several limitations. First, several factors limit the internal validity and interpretation of the findings. The single-arm pre–post design without a control group precludes causal attribution of the observed changes to MVP, as regression to the mean, natural temporal variation, expectancy effects, and other nonspecific influences cannot be excluded. Moreover, because MVP is a multicomponent intervention, the contribution of mindfulness practice or any other individual component cannot be determined. The health-related outcomes were assessed using self-report measures without complementary objective measures, and multiple outcomes were examined without adjustment for multiple comparisons; therefore, the observed pre–post changes, particularly those for exploratory outcomes, should be interpreted cautiously. Prior meditation experience and concurrent psychological or pharmacological treatment were not assessed, limiting our ability to account for their potential influence on the observed outcomes, particularly the CAMS-R results. The intervention was also delivered separately to three groups at different time points, but potential group- or time-related influences were not accounted for in the analyses.

Second, the generalizability of the findings is limited by the characteristics of the study sample and setting. Participants were self-selected volunteers recruited from a single public lifelong learning institution in Seoul, and the sample was predominantly female and relatively highly educated. The broad eligibility criteria and heterogeneity of participants’ physical and psychological concerns further limit generalization to more clearly defined populations. Participant compensation may have influenced retention, attendance, and satisfaction, and these feasibility and acceptability findings may not directly generalize to routine lifelong learning settings in which participants pay program fees rather than receive research participation incentives. In addition, no follow-up assessment was conducted beyond the end of the program; therefore, the longer-term sustainability of the observed changes remains unknown, particularly given that some participants reported difficulties maintaining mindfulness practice after program completion.

Third, several limitations of the feasibility and qualitative components should be considered. No *a priori* progression criteria were established for feasibility or acceptability, home-practice adherence was not systematically recorded, formal intervention fidelity was not independently assessed, no prespecified safety screening criteria were applied, and adverse events were not systematically monitored. Furthermore, the interview guide did not specifically address adverse experiences; therefore, the absence of such accounts should not be interpreted as evidence of program safety. The qualitative component was limited to self-selected program completers who received additional compensation for interview participation, which may have influenced their willingness to participate and potentially their responses; participants classified as dropouts were not interviewed. The first author served as the program facilitator, interviewer, and analyst, while members of the research team had previously developed the IVS and the Integrative Vitality Model. Although reflexivity and research-team discussions were incorporated into the analytic process, these overlapping roles and the pre-existing researcher–participant relationship may have influenced participants’ accounts and their interpretation of the data. The interview guide was not pilot-tested, and some questions were framed around participants’ experiences and perceived changes following the program, which may have influenced the direction of their responses. Furthermore, field notes, transcript return, and participant checking were not undertaken, and no formal assessment of data saturation or information power was conducted.

Future studies should employ controlled designs with prespecified outcomes and longer-term follow-up and include larger and more diverse samples across multiple settings, including greater representation of male participants. Future feasibility studies should also prospectively define progression criteria and incorporate systematic assessment of home-practice adherence, intervention fidelity, and safety. These methodological improvements would allow more rigorous evaluation of the efficacy of MVP and direct testing of the mechanisms proposed by the Integrative Vitality Model.

## 5 Conclusion

The MVP program showed preliminary feasibility and acceptability as a lifelong learning initiative for promoting health and vitality. Significant pre–post changes were observed in integrative vitality and subjective well-being, along with reductions in fatigue, depression, and anxiety. In the qualitative findings, participants described perceived changes in self-regulation, interpersonal relationships, and their sense of agency. Program retention, session attendance, and participant satisfaction provided additional information regarding the feasibility and acceptability of the program. These findings suggest that the MVP program may represent a practical lifelong learning intervention with potential relevance for public health promotion. Further controlled studies are needed to evaluate its efficacy.

## Data Availability

The raw data supporting the conclusions of this article will be made available by the authors, without undue reservation.

## References

[1] Juni MH. Ageing population: a public health implications. Int J Public Health Clin Sci. (2015) 2:1–5.

[2] United Nations. World Population Prospects 2022: Summary of Results. New York: United Nations (2022).

[3] O'Caoimh R Sezgin D O'Donovan MR Molloy DW Clegg A Rockwood K . Prevalence of frailty in 62 countries across the world: a systematic review and meta-analysis of population-level studies. Age Ageing. (2021) 50:96–104. doi: 10.1093/ageing/afaa219 33068107

[4] Soysal P Veronese N Thompson T Kahl KG Fernandes BS Prina AM . Relationship between depression and frailty in older adults: a systematic review and meta-analysis. Ageing Res Rev. (2017) 36:78–87. doi: 10.1016/j.arr.2017.03.005, 28366616

[5] Weiss CO. Frailty and chronic diseases in older adults. Clin Geriatr Med. (2011) 27:39–52. doi: 10.1016/j.cger.2010.08.003, 21093721

[6] Clarke DM Currie KC. Depression, anxiety and their relationship with chronic diseases: a review of the epidemiology, risk and treatment evidence. Med J Aust. (2009) 190:S54–60. doi: 10.5694/j.1326-5377.2009.tb02471.x, 19351294

[7] Boing AF Melo GR Boing AC Moretti-Pires RO Peres KG Peres MA. Association between depression and chronic diseases: results from a population-based study. Rev Saude Publica. (2012) 46:617–23. doi: 10.1590/S0034-89102012005000044, 22735271

[8] Korea Disease Control and Prevention Agency 2022 National Health Statistics (2023). Available online at: https://knhanes.kdca.go.kr/knhanes/sub04/sub04_04_01.do (Accessed October 20, 2024).

[9] Institute for Health Metrics and Evaluation (IHME) GBD Results (2021). Available online at: https://vizhub.healthdata.org/gbd-results/ (Accessed August 28, 2024).

[10] Cho SE Choi EY Oh YS Kim YS Kim SB. Investigating the predictors of frailty: an age-dependent analysis. Health Soc Welf Rev. (2017) 37:139–69. doi: 10.15709/hswr.2017.37.3.139

[11] Rudnicka E Napierała P Podfigurna A Męczekalski B Smolarczyk R Grymowicz M. The World Health Organization (WHO) approach to healthy ageing. Maturitas. (2020) 139:6–11. doi: 10.1016/j.maturitas.2020.05.018, 32747042 PMC7250103

[12] World Health Organization. WHO Clinical Consortium on Healthy Ageing 2018: Report of Consortium Meeting Held 11–12 December 2018 in Geneva, Switzerland. Geneva: World Health Organization (2019).

[13] Kellett J Sebat F. Make vital signs great again–a call for action. Eur J Intern Med. (2017) 45:13–9. doi: 10.1016/j.ejim.2017.09.018, 28941841

[14] Middleton A Fritz SL Lusardi M. Walking speed: the functional vital sign. J Aging Phys Act. (2015) 23:314–22. doi: 10.1123/japa.2013-0236, 24812254 PMC4254896

[15] Morone NE Weiner DK. Pain as the fifth vital sign: exposing the vital need for pain education. Clin Ther. (2013) 35:1728–32. doi: 10.1016/j.clinthera.2013.10.001, 24145043 PMC3888154

[16] Masciocchi E Maltais M El Haddad K Virecoulon Giudici K Rolland Y Vellas B Defining vitality using physical and mental well-being measures in nursing homes: a prospective study. J Nutr Health Aging (2020) 24:37–42. doi:doi: 10.1007/s12603-019-1285-8, 31886806 PMC6934632 31886806

[17] Prakash RS Voss MW Erickson KI Kramer AF. Physical activity and cognitive vitality. Annu Rev Psychol. (2015) 66:769–97. doi: 10.1146/annurev-psych-010814-015249, 25251492

[18] Ryan RM Frederick C. On energy, personality, and health: subjective vitality as a dynamic reflection of well-being. J Pers. (1997) 65:529–65. 10.1111/j.1467-6494.1997.tb00326.x 9327588

[19] Shapiro J Donaldson SI. The leader vitality scale: development, psychometric assessment, and validation. Front Psychol. (2022) 13:884672. doi: 10.3389/fpsyg.2022.884672, 35756249 PMC9226326

[20] Yoon SI Park HY Chung SY Kim JW. Development and validation of the integrative vitality scale. Front Public Health. (2024) 12:1452068. doi: 10.3389/fpubh.2024.1452068, 39624405 PMC11609079

[21] Arslan G. Loneliness, college belongingness, subjective vitality, and psychological adjustment during coronavirus pandemic: development of the college belongingness questionnaire. J Posit Sch Psychol. (2021) 5:17–31. doi: 10.47602/jpsp.v5i1.240

[22] Arslan G Yıldırım M Aytaç M. Subjective vitality and loneliness explain how coronavirus anxiety increases rumination among college students. Death Stud. (2022) 46:1042–51. doi: 10.1080/07481187.2020.1824204, 32998663

[23] Kubzansky LD Thurston RC. Emotional vitality and incident coronary heart disease: benefits of healthy psychological functioning. Arch Gen Psychiatry. (2007) 64:1393–401. doi: 10.1001/archpsyc.64.12.1393, 18056547

[24] Penninx BW Guralnik JM Bandeen-Roche K Kasper JD Simonsick EM Ferrucci L . The protective effect of emotional vitality on adverse health outcomes in disabled older women. J Am Geriatr Soc. (2000) 48:1359–66. doi: 10.1111/j.1532-5415.2000.tb02622.x, 11083309

[25] Kabat-Zinn J. Full Catastrophe Living. New York: Bantam (2013).

[26] Shapiro SL Carlson LE Astin JA Freedman B. Mechanisms of mindfulness. J Clin Psychol. (2006) 62:373–86. doi: 10.1002/jclp.20237, 16385481

[27] Cour PI Petersen M. Effects of mindfulness meditation on chronic pain: a randomized controlled trial. Pain Med. (2015) 16:641–52. doi: 10.1111/pme.12605, 25376753

[28] Rusch HL Rosario M Levison LM Olivera A Livingston WS Wu T . The effect of mindfulness meditation on sleep quality: a systematic review and meta-analysis of randomized controlled trials. Ann N Y Acad Sci. (2019) 1445:5–16. doi: 10.1111/nyas.13996, 30575050 PMC6557693

[29] McCartney M Nevitt S Lloyd A Hill R White R Duarte R. Mindfulness-based cognitive therapy for prevention and time to depressive relapse: systematic review and network meta-analysis. Acta Psychiatr Scand. (2021) 143:6–21. doi: 10.1111/acps.13242, 33035356

[30] Canby NK Cameron IM Calhoun AT Buchanan GM. A brief mindfulness intervention for healthy college students and its effects on psychological distress, self-control, meta-mood, and subjective vitality. Mindfulness. (2015) 6:1071–81. doi: 10.1007/s12671-014-0356-5

[31] Merriam SB Kee Y. Promoting community wellbeing: the case for lifelong learning for older adults. Adult Educ Q. (2014) 64:128–44. doi: 10.1177/0741713613513633

[32] Commission of the European Communities. Making a European Area of Lifelong Learning a Reality. Brussels: Commission of the European Communities (2001).

[33] Narushima M Liu J Diestelkamp N. Lifelong learning in active ageing discourse: its conserving effect on wellbeing, health and vulnerability. Ageing Soc. (2018) 38:651–75. doi: 10.1017/S0144686X16001136, 29551843 PMC5848758

[34] Yamashita T Bardo AR Liu D Yoo JW. Education, lifelong learning and self-rated health in later life in the USA. Health Educ J. (2019) 78:328–39. doi: 10.1177/0017896918809500

[35] Hammond C. Impacts of lifelong learning upon emotional resilience, psychological and mental health: fieldwork evidence. Oxf Rev Educ. (2004) 30:551–68. doi: 10.1080/0305498042000303008

[36] Benson H Klipper MZ. The Relaxation Response. New York: Morrow (1975).

[37] Selye H. The general adaptation syndrome and the diseases of adaptation. J Clin Endocrinol. (1946) 6:117–230. 10.1210/jcem-6-2-117 21025115

[38] American Psychiatric Association. Diagnostic and Statistical Manual of Mental Disorders: DSM-5. Washington, DC: American Psychiatric Association (2013).

[39] Lee MK Lee YH Park SH Sohn CH Jung YJ Hong SK . A standardization study of Beck depression inventory I-Korean version (K-BDI): reliability and factor analysis. Korean J Psychopathol. (1995) 4:77–95.

[40] Chalder T Berelowitz G Pawlikowska T Watts L Wessely S Wright D . Development of a fatigue scale. J Psychosom Res. (1993) 37:147–53. 10.1016/0022-3999(93)90081-p 8463991

[41] Lee JI Kwon JH. The effects of worry and emotional regulation on sleep and fatigue. Korean J Clin Psychol. (2008) 27:1–14. doi: 10.15842/kjcp.2008.27.1.001

[42] Lee Y Baek Y Park K Jin HJ Lee S. Development and validation of an instrument to measure the health status of healthy but unsatisfied people: Mibyeong index (未病 index). J Soc Prev Korean Med. (2016) 20:45–53.

[43] Suh EK Koo JS. A concise measure of subjective well-being (COMOSWB): scale development and validation. Korean J Soc Personal Psychol. (2011) 25:95–113. doi: 10.21193/kjspp.2011.25.1.006

[44] Cho YR. The reliability and validity of a Korean version of the cognitive and affective mindfulness scale-revised. Korean J Clin Psychol. (2009) 28:667–93. doi: 10.15842/kjcp.2009.28.3.002

[45] Braun V Clarke V. Using thematic analysis in psychology. Qual Res Psychol. (2006) 3:77–101. doi: 10.1191/1478088706qp063oa

[46] Terry G Hayfield N Clarke V Braun V. "Thematic analysis". In: Willig C Stainton-Rogers W, editors. The SAGE Handbook of Qualitative Research in Psychology, 2nd Edn. CA: SAGE Publications (2017). p. 17–37.

[47] Fetters MD Curry LA Creswell JW. Achieving integration in mixed methods designs—principles and practices. Health Serv Res. (2013) 48:2134–56. doi: 10.1111/1475-6773.12117, 24279835 PMC4097839

[48] Gamaiunova L Kreibig SD Dan-Glauser E Pellerin N Brandt PY Kliegel M. Effects of two mindfulness based interventions on the distinct phases of the stress response across different physiological systems. Biol Psychol. (2022) 172:108384. doi: 10.1016/j.biopsycho.2022.108384, 35753560

[49] Raugh IM Berglund AM Strauss GP. Implementation of mindfulness-based emotion regulation strategies: a systematic review and meta-analysis. Affect Sci. (2024) 6:171–200. doi: 10.1007/s42761-024-00281-x, 40094043 PMC11903998

[50] Xu JQ Tang YMJ Chen HYK. Impact of an online mindfulness-based program on wellbeing and trait mindfulness for research postgraduate students: a randomized-controlled trial. BMC Psychol. (2025) 13:28. doi: 10.1186/s40359-024-02233-3, 39794847 PMC11720815

[51] Garland EL Hanley AW Nakamura Y Barrett JW Baker AK Reese SE . Mindfulness-oriented recovery enhancement vs supportive group therapy for co-occurring opioid misuse and chronic pain in primary care: a randomized clinical trial. JAMA Intern Med. (2022) 182:407–17. doi: 10.1001/jamainternmed.2022.0033, 35226053 PMC8886485

[52] Liu W Yuan J Wu Y Xu L Wang X Meng J . A randomized controlled trial of mindfulness-based cognitive therapy for major depressive disorder in undergraduate students: dose-response effect, inflammatory markers and BDNF. Psychiatry Res. (2024) 331:115671. doi: 10.1016/j.psychres.2023.115671, 38101069

[53] Amaro H Black DS. Mindfulness-based intervention effects on substance use and relapse among women in residential treatment: a randomized controlled trial with 8.5-month follow-up period from the moment-by-moment in women's recovery project. Biopsychosoc Sci Med. (2021) 83:528–38. doi: 10.1097/PSY.0000000000000907, 34213858 PMC8257470

[54] Mellner C Osika W Niemi M. Mindfulness practice improves managers' job demands-resources, psychological detachment, work-nonwork boundary control, and work-life balance–a randomized controlled trial. Int J Workplace Health Manag. (2022) 15:493–514. doi: 10.1108/IJWHM-07-2021-0146

[55] MVC Team El Morr C Ritvo P Ahmad F Moineddin R. Effectiveness of an 8-week web-based mindfulness virtual community intervention for university students on symptoms of stress, anxiety, and depression: randomized controlled trial. JMIR Ment Health. (2020) 7:e18595. doi: 10.2196/18595, 32554380 PMC7395254

[56] Galla BM O'Reilly GA Kitil MJ Smalley SL Black DS. Community-based mindfulness program for disease prevention and health promotion: targeting stress reduction. Am J Health Promot. (2015) 30:36–41. doi: 10.4278/ajhp.131107-QUAN-567, 25162319

[57] Galante J Friedrich C Dawson AF Modrego-Alarcón M Gebbing P Delgado-Suárez I . Mindfulness-based programmes for mental health promotion in adults in nonclinical settings: a systematic review and meta-analysis of randomised controlled trials. PLoS Med. (2021) 18:e1003481. doi: 10.1371/journal.pmed.1003481, 33428616 PMC7799763

[58] Van Dam NT Earleywine M Danoff-Burg S. Differential item function across meditators and non-meditators on the five facet mindfulness questionnaire. Personal Individ Differ. (2009) 47:516–21. doi: 10.1016/j.paid.2009.05.005, 42574925

[59] Stjernswärd S Hansson L. A qualitative study of caregivers' experiences, motivation and challenges using a web-based mindfulness intervention. Community Ment Health J. (2020) 56:416–25. doi: 10.1007/s10597-019-00477-7, 31571086 PMC7056690

