# Supplementary material for: Feasibility and acceptability of a mindfulness-based vitality promotion program in a lifelong learning setting: a single-arm mixed-methods study
Source: Front Public Health. 2026 Oct 28;14:1990679. doi: 10.3389/fpubh.2026.1990679 (PMC13751955; doi:10.3389/fpubh.2026.1990679)
Supplement: Supplementary file 1 [file Table_1.docx]

# Supplementary Material 1(A). TREND Statement Checklist

Reporting checklist for nonrandomized evaluations of behavioral and public health interventions. Enter the manuscript page number(s) in the final column; use “N/A” where an item is not applicable to the single-arm design.

| **Paper section / topic** | **Item No.** | **Descriptor** | **Manuscript page(s)** |
| --- | --- | --- | --- |
| Title and Abstract | 1 | Information on how units were allocated to interventions; structured abstract recommended; information on target population or study sample. | p.1 |
| Introduction – Background | 2 | Scientific background and explanation of rationale; theories used in designing behavioral interventions. | p.2-3 |
| Methods – Participants | 3 | Eligibility criteria; recruitment method/sampling; recruitment setting; settings and locations of data collection. | p.3-4 |
| Methods – Interventions | 4 | Intervention details: content, delivery method, unit of delivery, deliverer, setting, exposure quantity/duration, time span, and activities to increase compliance/adherence (e.g., incentives). | p.5-6 |
| Methods – Objectives | 5 | Specific objectives and hypotheses. | p.3 |
| Methods – Outcomes | 6 | Clearly defined primary and secondary outcomes; data collection methods/measurement quality; information on validated instruments. | p.3, 6-8 |
| Methods – Sample Size | 7 | How sample size was determined and, when applicable, interim analyses and stopping rules. | p.4 |
| Methods – Assignment Method | 8 | Unit of assignment; method used to assign units to conditions; methods to minimize bias due to non-randomization. | N/A |
| Methods – Blinding (masking) | 9 | Whether participants, intervention providers, and outcome assessors were blinded; if applicable, how blinding was accomplished and assessed. | N/A |
| Methods – Unit of Analysis | 10 | Smallest unit analyzed; if different from assignment unit, analytical method used to account for this. | p.8 |
| Methods – Statistical Methods | 11 | Statistical methods for primary outcomes and additional analyses; missing-data imputation methods; statistical software. | p.8 |
| Results – Participant Flow | 12 | Participant flow through enrollment, assignment/allocation, intervention exposure, follow-up, and analysis; protocol deviations and reasons. A flow diagram is strongly recommended. | p.4-5; Figure 1 |
| Results – Recruitment | 13 | Dates defining recruitment and follow-up periods. | p.5 |
| Results – Baseline Data | 14 | Baseline demographic/clinical characteristics; relevant prevention characteristics; comparison of lost-to-follow-up vs retained participants; comparison with target population, as applicable. | p.9; Table 3; Baseline characteristics reported; no completer–non-completer comparison conducted. |
| Results – Baseline Equivalence | 15 | Study-group equivalence at baseline and methods used to control baseline differences, as applicable. | N/A |
| Results – Numbers Analyzed | 16 | Number of participants included in each analysis; analysis strategy (e.g., intention-to-treat) and handling of non-compliers. | p.8-10; Table 4; Supplementary Materials 3-4 |
| Results – Outcomes and Estimation | 17 | Results for primary/secondary outcomes; effect sizes and confidence intervals; null/negative findings; prespecified causal-pathway results, if any. | p.10; Table 4 |
| Results – Ancillary Analyses | 18 | Other analyses, including subgroup/restricted analyses, indicating prespecified vs exploratory. | p.8, 10; Supplementary Materials 3-4 |
| Results – Adverse Events | 19 | Important adverse events or unintended effects. | Not assessed (p.4, 16-17). |
| Discussion – Interpretation | 20 | Interpretation considering hypotheses, potential bias, imprecision, multiple analyses, and limitations; mechanisms/alternative explanations; implementation success/barriers and fidelity; research/program/policy implications. | p.14-17 |
| Discussion – Generalizability | 21 | External validity considering population, intervention, follow-up, incentives, compliance, sites/settings, and contextual issues. | p.16-17 |
| Discussion – Overall Evidence | 22 | General interpretation in the context of current evidence and theory. | p.14-17 |

# Supplementary Material 1(B). COREQ 32-item Checklist

Consolidated Criteria for Reporting Qualitative Research (COREQ) for interviews and focus groups. Enter the manuscript page number(s) in the final column.

| **Domain / section** | **Item No.** | **Item** | **Guide question / description** | **Manuscript page(s)** |
| --- | --- | --- | --- | --- |
| Domain 1: Research team and reflexivity – Personal characteristics | 1 | Interviewer/facilitator | Which author(s) conducted the interview or focus group? | p.5 |
|  | 2 | Credentials | What were the researcher’s credentials (e.g., PhD, MD)? | p.5 |
|  | 3 | Occupation | What was the researcher’s occupation at the time of the study? | p.5 |
|  | 4 | Gender | Was the researcher male or female? | p.5 |
|  | 5 | Experience and training | What experience or training did the researcher have? | p.5 |
| Domain 1: Research team and reflexivity – Relationship with participants | 6 | Relationship established | Was a relationship established prior to study commencement? | p.5 |
|  | 7 | Participant knowledge of interviewer | What did participants know about the researcher (e.g., personal goals, reasons for doing the research)? | p.4-5 |
|  | 8 | Interviewer characteristics | What interviewer/facilitator characteristics were reported (e.g., bias, assumptions, reasons and interests in the research topic)? | p.5, 9 |
| Domain 2: Study design – Theoretical framework | 9 | Methodological orientation and theory | What methodological orientation was stated to underpin the study (e.g., grounded theory, discourse analysis, ethnography, phenomenology, content analysis)? | p.8 |
| Domain 2: Study design – Participant selection | 10 | Sampling | How were participants selected (e.g., purposive, convenience, consecutive, snowball)? | p.5 |
|  | 11 | Method of approach | How were participants approached (e.g., face-to-face, telephone, mail, email)? | p.5 |
|  | 12 | Sample size | How many participants were in the study? | p.10; Figure 1 |
|  | 13 | Non-participation | How many people refused to participate or dropped out? Reasons? | p.5, 10; Figure 1 |
| Domain 2: Study design – Setting | 14 | Setting of data collection | Where was the data collected (e.g., home, clinic, workplace)? | p.5 |
|  | 15 | Presence of non-participants | Was anyone else present besides participants and researchers? | p.5 |
|  | 16 | Description of sample | What are the important characteristics of the sample (e.g., demographic data, date)? | Table 5 |
| Domain 2: Study design – Data collection | 17 | Interview guide | Were questions, prompts, or guides provided? Was the guide pilot tested? | p.5; Table 1; not pilot-tested. |
|  | 18 | Repeat interviews | Were repeat interviews carried out? If yes, how many? | p.5 |
|  | 19 | Audio/visual recording | Did the research use audio or visual recording to collect data? | p.5 |
|  | 20 | Field notes | Were field notes made during and/or after the interview or focus group? | Not performed. |
|  | 21 | Duration | What was the duration of the interviews or focus groups? | p.5 |
|  | 22 | Data saturation | Was data saturation discussed? | Not performed. No formal assessment of data saturation was undertaken; interview participation was based on voluntary participation among program completers. |
|  | 23 | Transcripts returned | Were transcripts returned to participants for comment and/or correction? | Not performed. |
| Domain 3: Analysis and findings – Data analysis | 24 | Number of data coders | How many data coders coded the data? | p.9 |
|  | 25 | Description of the coding tree | Did the authors provide a description of the coding tree? | Table 6 |
|  | 26 | Derivation of themes | Were themes identified in advance or derived from the data? | p.9 |
|  | 27 | Software | What software, if applicable, was used to manage the data? | Not used. (p.9) |
|  | 28 | Participant checking | Did participants provide feedback on the findings? | Not performed. |
| Domain 3: Analysis and findings – Reporting | 29 | Quotations presented | Were participant quotations presented to illustrate themes/findings, and was each quotation identified (e.g., participant number)? | p.10-13 |
|  | 30 | Data and findings consistent | Was there consistency between the data presented and the findings? | p.10-13; Table 6 |
|  | 31 | Clarity of major themes | Were major themes clearly presented in the findings? | p.10-13; Table 6 |
|  | 32 | Clarity of minor themes | Was there a description of diverse cases or discussion of minor themes? | p.10-13; Table 6 |

**Source notes:** TREND: Des Jarlais DC, Lyles C, Crepaz N, and the TREND Group (2004). COREQ: Tong A, Sainsbury P, Craig J (2007). Checklist wording has been formatted for supplementary-file completion.
