# Supplementary material for: Feasibility and acceptability of a mindfulness-based vitality promotion program in a lifelong learning setting: a single-arm mixed-methods study
Source: Front Public Health. 2026 Oct 28;14:1990679. doi: 10.3389/fpubh.2026.1990679 (PMC13751955; doi:10.3389/fpubh.2026.1990679)
Supplement: Supplementary file 2 [file Table_2.docx]

# Supplementary Material 2. Satisfaction Survey

The satisfaction survey was administered in Korean. The original Korean items and their English translations are presented below.

**A. Quantitative items**

Participants rated each item on a 5-point Likert scale ranging from 0 (strongly disagree) to 4 (strongly agree).

| **No.** | **Original Korean item** | **English translation** |
| --- | --- | --- |
| 1 | 프로그램에 전반적으로 만족하였습니까? | Overall, were you satisfied with the program? |
| 2 | 프로그램의 구성과 내용은 만족스러웠습니까? | Were you satisfied with the structure and content of the program? |
| 3 | 프로그램의 장소는 만족스러웠습니까? | Were you satisfied with the program setting? |
| 4 | 프로그램의 총 기간 및 시간은 적절합니까? | Were the overall program duration and session length appropriate? |
| 5 | 다음에도 프로그램에 참여할 의사가 있습니까? | Would you be willing to participate in the program again? |
| 6 | 지인에게 프로그램을 추천할 의사가 있습니까? | Would you be willing to recommend the program to others? |

**B. Open-ended questions**

| **No.** | **Original Korean item** | **English translation** |
| --- | --- | --- |
| 1 | 본 프로그램에서 만족스러웠던 점은 무엇인지 구체적으로 기술해주시기 바랍니다. | Please describe in detail what you found satisfactory about this program. |
| 2 | 본 프로그램에서 개선되어야 할 점은 무엇인지 구체적으로 기술해주시기 바랍니다. | Please describe in detail any aspects of this program that could be improved. |
| 3 | 기타 소감이나 바라는 점을 자유롭게 기술해주시기 바랍니다. | Please freely describe any additional comments or suggestions regarding the program. |
