# Supplementary material for: Feasibility and acceptability of a mindfulness-based vitality promotion program in a lifelong learning setting: a single-arm mixed-methods study
Source: Front Public Health. 2026 Oct 28;14:1990679. doi: 10.3389/fpubh.2026.1990679 (PMC13751955; doi:10.3389/fpubh.2026.1990679)
Supplement: Supplementary file 3 [file Table_3.docx]

| **Supplementary Material 3** Wilcoxon signed-rank sensitivity analyses of changes in study outcomes | | | | |
| --- | --- | --- | --- | --- |
| **Variable** | **Baseline**  **(T0)** | **Post-intervention**  **(T1)** | ***Z*** | ***p*** |
| IVS | 47.00 [37.00–59.00] | 62.00 [47.00–71.00] | –4.450 | <0.001 |
| IVS-P | 20.00 [17.00–29.00] | 31.00 [22.00–35.00] | –4.707 | <0.001 |
| IVS-E | 28.00 [19.00–34.00] | 31.00 [25.00–38.00] | –3.275 | 0.001 |
| BDI | 17.00 [9.00–20.00] | 10.00 [5.00–16.00] | –4.191 | <0.001 |
| FS | 34.00 [29.00–40.00] | 28.00 [22.00–35.00] | –4.059 | <0.001 |
| MBI | 62.00 [49.00–81.00] | 48.00 [36.00–66.00] | –2.774 | 0.006 |
| MBI-fatigue | 10.00 [8.00–13.00] | 8.00 [6.00–11.00] | –2.407 | 0.016 |
| MBI-pain | 10.00 [6.00–12.00] | 8.00 [4.00–11.00] | –1.971 | 0.049 |
| MBI-sleep | 9.00 [6.00–14.00] | 8.00 [5.00–12.00] | –2.022 | 0.043 |
| MBI-digestion | 6.00 [3.00–11.00] | 6.00 [3.00–10.00] | –0.680 | 0.496 |
| MBI-depression | 9.00 [4.00–13.00] | 6.00 [3.00–10.00] | –3.113 | 0.002 |
| MBI-anger | 7.00 [5.00–10.00] | 6.00 [4.00–8.00] | –1.625 | 0.104 |
| MBI-anxiety | 9.00 [6.00–13.00] | 7.00 [4.00–10.00] | –3.011 | 0.003 |
| COMOSWB | 15.50 [7.50–23.25] | 23.00 [10.00–28.00] | –3.757 | <0.001 |
| CAMS-R | 27.00 [23.00–31.00] | 29.00 [26.00–31.00] | –1.393 | 0.164 |
| IVS, Integrative Vitality Scale; IVS-P, physical vitality subscale; IVS-E, psychological vitality subscale; BDI, Korean version of the Beck Depression Inventory; FS, Fatigue Scale; MBI, Mibyeong Index; COMOSWB, Concise Measure of Subjective Well-Being; CAMS-R, Korean version of the Cognitive and Affective Mindfulness Scale-Revised  Values are presented as median [25^th^–75^th^ percentile].  Analyses included 35 participants for all outcomes except COMOSWB (*n* = 34). Cases with zero differences between T0 and T1, including the four program dropouts whose baseline values were carried forward, were excluded from the calculation of the Wilcoxon signed-rank statistic. The sign of the Z statistic does not necessarily indicate the direction of change, which should be interpreted based on the descriptive statistics at T0 and T1. | | | | |
