# Supplementary material for: Feasibility and acceptability of a mindfulness-based vitality promotion program in a lifelong learning setting: a single-arm mixed-methods study
Source: Front Public Health. 2026 Oct 28;14:1990679. doi: 10.3389/fpubh.2026.1990679 (PMC13751955; doi:10.3389/fpubh.2026.1990679)
Supplement: Supplementary file 4 [file Table_4.docx]

| **Supplementary Material 4** Complete-case sensitivity analyses of changes in study outcomes among program completers | | | | | | |
| --- | --- | --- | --- | --- | --- | --- |
| **Variable** | **Baseline**  **(T0)** | **Post-intervention**  **(T1)** | **Mean change ± SD [95% CI]** | ***t*** | ***p*** | ***\|d*_z_*\|*** |
| IVS | 49.48 ± 14.74 | 61.71 ± 14.32 | 12.23 ± 11.36 [8.06, 16.39] | 5.99 | <0.001 | 1.08 |
| IVS-P | 22.52 ± 7.30 | 30.06 ± 7.16 | 7.55 ± 6.06 [5.33, 9.77] | 6.94 | <0.001 | 1.25 |
| IVS-E | 26.97 ± 8.69 | 31.65 ± 8.20 | 4.68 ± 6.92 [2.14, 7.22] | 3.76 | <0.001 | 0.68 |
| BDI | 15.55 ± 7.97 | 10.42 ± 8.46 | −5.13 ± 5.55 [−7.17, −3.09] | −5.14 | <0.001 | 0.92 |
| FS | 33.16 ± 7.35 | 27.46 ± 6.53 | −5.70 ± 6.18 [−7.97, −3.43] | −5.14 | <0.001 | 0.92 |
| MBI | 63.60 ± 23.18 | 51.39 ± 20.97 | −12.21 ± 21.11 [−19.95, −4.46] | −3.22 | 0.003 | 0.58 |
| MBI-fatigue | 9.80 ± 3.67 | 7.88 ± 2.77 | −1.92 ± 4.16 [−3.45, −0.39] | −2.57 | 0.015 | 0.46 |
| MBI-pain | 9.75 ± 4.69 | 7.78 ± 3.85 | −1.97 ± 5.49 [−3.98, 0.05] | −2.00 | 0.055 | 0.36 |
| MBI-sleep | 10.20 ± 4.17 | 8.52 ± 4.33 | −1.69 ± 4.74 [−3.42, 0.05] | −1.98 | 0.057 | 0.36 |
| MBI-digestion | 6.81 ± 4.44 | 6.32 ± 4.11 | −0.48 ± 3.40 [−1.73, 0.76] | −0.79 | 0.436 | 0.14 |
| MBI-depression | 9.29 ± 4.91 | 6.82 ± 4.39 | −2.47 ± 4.24 [−4.02, −0.92] | −3.25 | 0.003 | 0.58 |
| MBI-anger | 8.01 ± 4.28 | 6.42 ± 3.38 | −1.59 ± 4.39 [−3.20, 0.02] | −2.02 | 0.052 | 0.36 |
| MBI-anxiety | 9.74 ± 5.20 | 7.65 ± 4.50 | −2.09 ± 3.87 [−3.51, −0.67] | −3.01 | 0.005 | 0.54 |
| COMOSWB | 15.75 ± 9.15 | 21.50 ± 10.69 | 5.75 ± 7.61 [2.90, 8.59] | 4.14 | <0.001 | 0.75 |
| CAMS-R | 27.39 ± 6.24 | 28.58 ± 5.85 | 1.19 ± 4.59 [−0.49, 2.88] | 1.45 | 0.158 | 0.26 |
| IVS, Integrative Vitality Scale; IVS-P, physical vitality subscale; IVS-E, psychological vitality subscale; BDI, Korean version of the Beck Depression Inventory; FS, Fatigue Scale; MBI, Mibyeong Index; COMOSWB, Concise Measure of Subjective Well-Being; CAMS-R, Korean version of the Cognitive and Affective Mindfulness Scale-Revised  Mean change was calculated as T1 − T0. Analyses included 31 program completers for all outcomes except COMOSWB (*n* = 30). | | | | | | |
